# Effect of an Ultra-Diluted Complex on Health, Growth Performance, and Blood Parameters of Pre-Weaned Dairy Calves

**DOI:** 10.3390/vetsci12020128

**Published:** 2025-02-05

**Authors:** Mellory M. Martins, Thiago H. da Silva, Andre S. V. Palma, Bruna L. de Noronha, Emanuel R. Lemos, Iuli C. S. B. Guimarães, Nara. R. B. Cônsolo, Arlindo S. Netto

**Affiliations:** 1 Department of Animal Science, College of Animal Science and Food Engineering, University of São Paulo, Pirassununga 13635-900, SP, Brazil; dasilvath2@gmail.com (T.H.d.S.); bruna.noronha@usp.br (B.L.d.N.); saranetto@usp.br (A.S.N.); 2 Department of Animal Nutrition and Production, School of Veterinary Medicine and Animal Science, University of São Paulo, Pirassununga 13635-900, SP, Brazil

**Keywords:** acute phase protein, dairy calf, health, homeopathy, Holstein calves

## Abstract

This study aimed to evaluate the effects of an ultra-diluted complex on the health status, growth performance, and blood parameters of dairy calves during the preweaning phase in a double-blind, placebo-controlled trial. During a 75-period trial, thirty-four Holstein newborn calves at the second day of life were completely randomized individually into two treatments: (1) control (CON), oral saline supplementation (5 mL/d); and (2) oral supplementation with 5 mL/d of a ultra-diluted complex (UD): Sulfur: $10^{60}$ + Viola tricolor: $10^{14}$ + Caladium seguinum: $10^{30}$ + Zincum oxydatum: $10^{30}$ + Phosphorus: $10^{60}$ + Cardus marianus: $10^{60}$ + Colibacillinum: $10^{30}$ + Podophyllum: $10^{30}$ + Vehicle: alcohol. Free access to water and a starter was ensured during the trial period. Feed intake and fecal and health scores were monitored daily. Calves were weighed and measured weekly. Blood samples were collected at enrollment on the first day and every 21 days until the 75th day, 2 h after morning milk feeding, and tested for hematology, blood urea nitrogen, aspartate aminotransferase, gamma-glutamyl transferase, total protein albumin, globulin, and creatinine. At 70 days of age, calves were disbudded following the standard management practices of the farm. The inflammatory profile, including Immunoglobulin A, Immunoglobulin G, ceruloplasmin, transferrin, albumin, and haptoglobin, was quantified in serum samples. The ultra-diluted complex was ineffective in reducing the incidence of diseases, starter feed intake, daily weight gain, and body measurements and inflammatory profile. Blood parameters were also not affected. Thus, the ultra-diluted complex was inefficient in reducing the incidence of diseases and did not affect the performance and metabolites of pre-weaned Holstein calves.

## Simple Summary

This study investigated the effects of an ultra-diluted complex on the health, growth, and blood markers of dairy calves during the pre-weaning period. Thirty-four newborn Holstein calves were randomly divided into two groups: one received a placebo, and the other received an ultra-diluted complex. The calves were fed specific amounts of milk replacer daily until they were weaned at 75 days old and had constant access to water and starter feed. We monitored their food intake, health, and weight, and took blood samples every three weeks. The results showed that the ultra-diluted complex did not reduce disease incidence or improve growth and health compared to the saline solution. Our findings suggest that this ultra-diluted complex, at the given dosage, was not effective in enhancing the health or performance of dairy calves. More research is needed to explore the potential benefits of such products for farm animals.

## 1. Introduction

The pre-weaned dairy calves generally undergo a period of great challenges, which can be intensified by the type of production system and the management strategies adopted. Calves are exposed to various stress factors in a short period. Among these factors is birth, especially for calves born from dystocic deliveries or with failure in passive immunity transfer, which presents higher morbidity and mortality rates [1,2,3]. Other challenges include transportation, disbudding, weaning, and exposure to pathogens. These factors, along with the development of calves’ immunocompetence, can contribute to high morbidity and mortality rates in calf rearing, compromising animal welfare and the profitability of dairy farms [2].

Immunocompetence is defined as the ability to defend against potentially harmful microorganisms and parasites [4,5,6,7]. Thus, strategies aimed at increasing the immunocompetence of calves are highly desirable, as they have the potential to improve the productivity of the production system [7]. Calves that experience health issues such as diarrhea and pneumonia during their early life stages often exhibit reduced weight gain and lower milk production during their first lactation [8,9]. Reducing morbidity rates also leads to a reduction in the use of antimicrobials, an important goal considering the growing concern about antimicrobial resistance, which represents a public health issue [10].

Several approaches have been studied to improve the immunocompetence of dairy calves [11]. Many focus on natural compounds, which have received considerable attention due to the global trend of reducing antimicrobial use in livestock production systems [10]. In this context, the use of ultra-diluted compounds becomes a promising strategy.

Ultra-diluted compounds, also known as homeopathic compounds, were originally proposed in 1796 by Samuel Hahnemann and are based on the administration of highly diluted substances that, in healthy individuals, would induce symptoms similar to those of the disease to be treated. In sick individuals, these substances could alleviate similar symptoms [12].

Homeopathy traditionally focuses on treating individuals, rather than herds. In contrast, the concept of homotoxicology offers an alternative therapy that utilizes homeopathically diluted remedies to eliminate toxins from the body. The foundation of homotoxicology, as proposed by Hans Reckeweg, is based on three key principles: prevention of additional homotoxicological challenges, elimination of homotoxins, and regulation of existing “homotoxicoses”. Medications commonly used in this approach are homeopathic complexes/ultra-diluted compounds. Proponents of homotoxicology argue that these complexes activate the “greater defense system”, a coordinated response involving the neurological, endocrine, immunological, metabolic, and connective tissue systems, which can lead to symptoms and facilitate the excretion of homotoxins [13]. As such, homotoxicology may be considered a more plausible alternative to traditional homeopathy for herd application, despite its efficacy still being under investigation [13,14].

The mechanisms of action of ultra-diluted compounds and the theoretical basis are not fully understood, and there are significant conflicting results and a limited number of high-rigor scientific studies. However, previous research has revealed that the use of ultra-diluted compounds has the potential to reduce the occurrence of digestive disorders and the numb.er of days affected by tick-borne diseases in weaned calves, as well as minimize the risk of heifer culling [15].

Aiming to contribute to this gap, the compound used in this study was the same as that evaluated in the previous study [15], which showed potential for reducing digestive disorders, indicating a promising strategy considering that diarrhea in calves is one of the main causes of mortality [2,16]. We hypothesize that the administration of ultra-diluted compounds would decrease the risk of disease incidence, consequently resulting in better performance among pre-weaned dairy calves.

## 2. Materials and Methods

All experimental procedures involving animal care were conducted according to the Institutional Animal Care and Use Committee Guidelines (IACUC #2946311019) of the College of Animal Science and Food Engineering of the University of Sao Paulo. This experiment was conducted on a high-yielding commercial dairy farm in southeast Minas Gerais in Carmo do Rio Claro state, Brazil, from January to April 2019.

### 2.1. Experimental Design, Treatment Allocation, and Inclusion Criteria

Thirty-four female Holstein calves (BW = 37.27 kg ± 3.83; mean ± SD) were enrolled in a double-blind, randomized, placebo-controlled trial. Calves were allocated to the treatments based on meeting the inclusion criteria, which included: efficiency of passive immune transfer, no history of dystocia births, and no symptoms of diarrhea or pneumonia. To ensure randomization, the RANDBETWEEN function in Excel was used to assign each calf to a treatment group as it became eligible for the study. The two treatments were: control (CON), with oral supplementation of 5 mL of saline solution (*n* = 17); and ultra-diluted complex (UD), with oral supplementation of 5 mL of an ultra-diluted complex (Sulphur: 10^−60^ (chemical element, mineral) + *Viola tricolor*: 10^−14^ (plant extract) + *Caladium seguinum*: 10^−30^ (plant extract) + *Zincum oxydatum:* 10^−30^ (zinc oxide, chemical compound) + Phosphorus: 10^−60^ (chemical element) + *Cardus marianus* 10^−60^ (plant extract): + *Colibacillinum*: 10^−30^ (prepared from strains of *Escherichia coli,* bacterial origin) + *Podophyllum*:10^−30^ (plant extract) + Vehicle: alcohol, Real H Company, Campo Grande, MS, Brazil; (*n* = 17)). The treatments were initiated at two days of age and administered orally once daily for 75 days using a 10 mL disposable syringe, after the first milk replacer feeding.

The study included only calves with brix refractometer readings ≥ 8.4%, considered to have efficient passive immune transfer, as animals with passive immune transfer failure exhibit higher rates of morbidity and mortality [17]. Only calves born from unassisted parturition were enrolled. Dystocia is defined as delayed or difficult parturition [18]. In this study, dystocia was classified according to a scale adapted from [3]. A dystocia score of 1 was assigned to births requiring no assistance. A score of 2 was to assigned to calving events that required intervention by one person without the use of mechanical assistance. A score of 3 was assigned to any calving event that required the assistance of 2 or more people. Only animals with a score of 1 (i.e., no intervention during delivery) were included in the study, as calves born from dystocia have higher rates of morbidity and mortality [3]. This study did not include calves exhibiting clinical signs associated with diarrhea, characterized by a fecal score of 3 or higher, as outlined by [19], nor did it include animals with bovine respiratory disease (BRD).

A blood sample was collected from the jugular vein 48 h after the initial colostrum feeding, and the serum was analyzed using a refractometer [20]. No significant differences in passive immune transfer were found between the groups. The average Brix values for the control and supplemented groups were 9.29 ± 0.19 and 9.08 ± 0.19, respectively [20].

### 2.2. Animals and Housing

**Figure 1:**
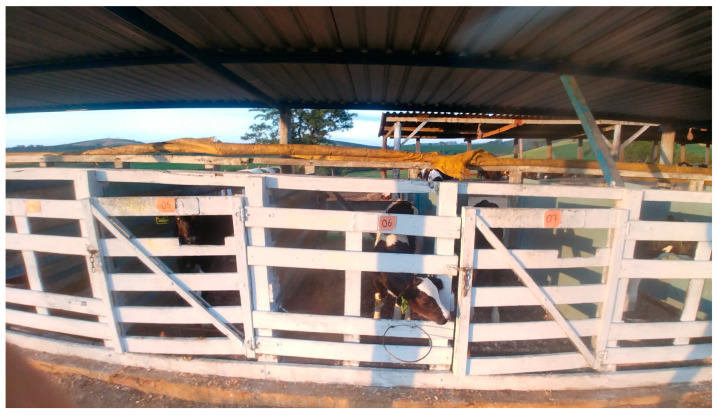
Individual housing of calves from 1 to 30 days of age.

**Figure 2:**
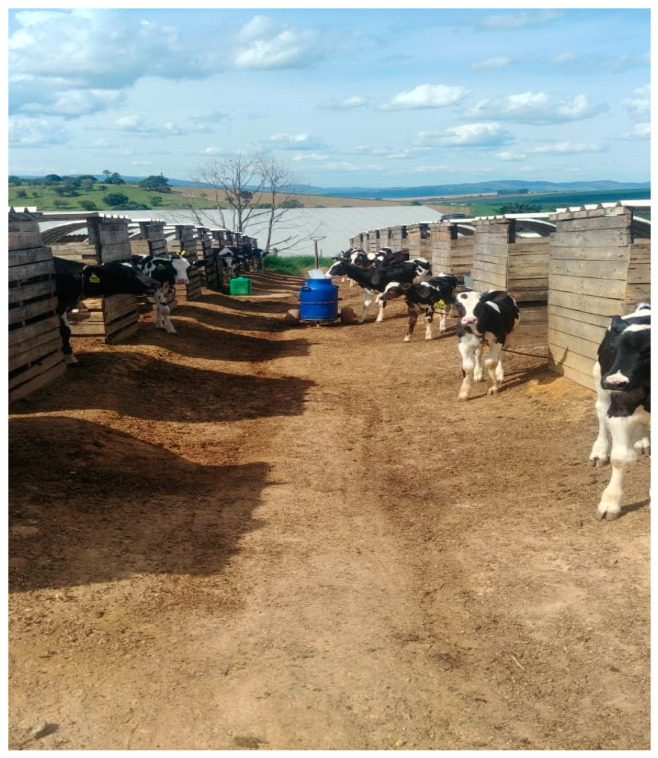
Individual housing of calves from 31 days of age to 75 days.

Calves were separated from their dams immediately after birth and were individually housed wood shelters bedded with wood shavings, called nursery pens (Figure 1), each with an average area of 3 square meters. When they reached 30 days of age, they were transferred to another individual housing system where they were contained by a string belt attached to a string, which provided them with sufficient space for walking but prevented any physical contact with other animals (Figure 2).

To prevent navel infection, the umbilical cord was dipped in a 7% iodine alcoholic solution. The calves were fed with at least 10% of their body weight in colostrum within the first 6 h of life. The quality of the colostrum was assessed using a Brix refractometer (Sper Scientific 300001 Refractometer, Brix: 0–32%). Only high-quality colostrum (≥60 mg/L of IgG) was offered to the calves. When the fresh colostrum did not meet the quality and/or volume requirements, colostrum from the farm’s colostrum bank was thawed and provided to the neonate. A blood sample was collected from the jugular vein 48 h after the first feeding, and the serum from this sample was analyzed using a Brix refractometer to evaluate the efficiency of passive immunity transfer, which was considered adequate with a refractometer reading ≥8.4%.

Calves were fed twice daily (07:00 h and 16:00 h). For the first 10 days, each calf received 4 L/day of whole milk using a bucket-teat container. After this period, they were offered 6 L/day of milk replacer (Natimilk, Auster^®^) containing 18% fat and 23% protein in a plastic bucket until the 70th day of the study. Subsequently, the liquid feeding was gradually reduced by 1 L per day until weaning on the 75th day. In addition, commercial starter feed and water were provided ad libitum from birth. Starter concentrate was offered daily in the morning, and the leftover orts from the previous day were collected and weighed on a digital scale to calculate the daily intake.

### 2.3. Chemical Analysis

Starter concentrate samples were collected biweekly and analyzed for the determination of the partial dry matter and subsequently ground in a Willey mill through a 1 mm sieve. Dry matter (105 °C; AOAC 950.15), ash (AOAC, 942.05), and ether extract (AOAC 920.39) contents were obtained according to [21]. Crude protein content (AOAC, 984.13) was obtained by multiplying the total nitrogen content by 6.25 using the Dumas combustion method, according to [21]. Neutral (NDF) and acid (ADF) detergent fibers were obtained as described by [22]. Total digestible nutrients (TDN) were determined according to [23], total carbohydrates were calculated according to the methodology of [24], and the levels of non-fibrous carbohydrates were calculated as proposed by [25].

### 2.4. Calf Growth and Performance

Body weight and body measurements were assessed at birth and then weekly until the end of the study (75 days after birth) using calibrated weight tapes measuring heart girth circumference [26]. These measurements were used to calculate the average daily gain (ADG). Also, wither height (cm) and hearth girth (cm) were assessed using a hipometer. The average daily withers height (cm/d), body depth (cm/d), and hearth girth (cm/d) gains were calculated by the difference between final and initial measures divided by the period in days. All measurements were performed by the same person to minimize experimental errors.

### 2.5. Case Definition

The fecal score was monitored daily for feces fluidity (1 = normal and firm; 2 = loose but with a general generally healthy aspect; 3 = very loose, no watery separation; and 4 = watery [20]); fecal scores ≥3 were considered diarrhea when they occurred for 2 or more consecutive days. Oral rehydration solution (2 L/d, regardless of diarrhea severity) was provided between milk feedings when the fecal score was ≥3 until the score was ≤2. The hydration solution used was the Glutellac^®^, a commercial electrolyte solution, based on sodium acetate 18.3 g, glucose 19.5 g, sodium and potassium chloride 3.2 g, flavoring 0.015 g, and sodium diacetate 1.6 g in a blister of 50 mL (Bayer SA, São Paulo, Brazil). Assessments of calves’ health, as well as diagnoses, interventions, and antimicrobial stewardship, were performed daily.

Respiratory problems were characterized by fever (rectal temperature ≥ 39.4 °C) associated with increased respiratory frequency (>60 inspirations per minute) and the presence of increased lung noise at auscultation. Data regarding the risk of disease incidence and treatment events were recorded daily by the research team. Diagnostic and antibiotic therapy was administered by personnel responsible for the farm’s health management, who were blinded to the treatments. The resolution of diseases was determined based on the restoration of the animal’s clinical status within the physiological reference limits. For enteric and respiratory diseases, improvements in the recorded scores and parameters were also considered. The end of the disease was considered when the animal’s clinical status returned to the physiological reference limits, and in the case of enteric and respiratory diseases, improvement in the described scores and parameters was also considered.

### 2.6. Blood Collection and Evaluation

Blood samples (8 mL) were collected from the jugular vein every 21 days using Vacutainer tubes with EDTA. After collection, the tubes were immediately placed into a cooler with iced water and transported to a commercial laboratory (EXAME, Clinical Analysis, Carmo do Rio Claro, SP, Brazil). Red blood cell (RBC) and white blood cell (WBC) count, hemoglobin (Hb) level, packed cell volume, mean corpuscular volume, mean corpuscular Hb (MCH), and MCH concentration measurements were obtained using manual techniques. WBC subtypes (neutrophils (band), neutrophils (segmented), lymphocytes, monocytes, eosinophils, and basophils were assessed by evaluating blood smears using a light microscope. Blood samples were also collected in tubes without anticoagulant and centrifuged at 2500× *g* for 15 min. The serum was sampled and stored at −20 °C for subsequent analysis of total protein albumin and globulin, aspartate aminotransferase, gamma-glutamyl transferase, creatinine, urea, and total iron binding capacity using commercial tests (Labtest, Brazil). Blood metabolites were measured using a biochemical analyzer (Mindray, BS120, Shenzhen, China).

### 2.7. Challenge: Disbudding and Inflammatory Profile Analysis

A total of 24 calves, at 70 days of age, were disbudded following the standard management practices of the farm. The animals were restrained and asepsis of the horn buds was performed, followed by cauterization with a hot iron. Before the disbudding procedure, a clinical screening was performed by a trained veterinarian, and the Calf Health Score [19,27,28] was assessed. Animals that presented with fever or alterations in health scores were not subjected to the procedure to avoid potential interactions with the evaluation responses, inflammatory profile, and temperature. Blood samples were collected via jugular vein puncture and stored in specific tubes. Samples in tubes with a clot activator were centrifuged at 2000× *g* for 15 min, identified, and stored at −20 °C for inflammatory profile analysis.

The inflammatory profile, including immunoglobulin A, immunoglobulin G, ceruloplasmin, transferrin, albumin, and haptoglobin, was quantified in serum samples. Total serum protein concentration was assessed utilizing the biuret method with a commercial kit (Total Protein; Labtest, Lagoa Santa, Brazil). Protein fractions were separated through acrylamide gel electrophoresis containing sodium dodecyl sulfate (SDS-PAGE), following the procedure described by [29]. Post-fractionation, the gel was stained for 10 min in a Coomassie blue solution (comprising 50.0% methanol, 40.0% water, 9.75% glacial acetic acid, and 0.25% Coomassie blue). Subsequently, the gel was immersed in a 7.0% acetic acid solution to eliminate excess dye until distinct protein fractions were visible. The proteins were then determined using a computerized densitometer.

Blood samples for the inflammatory profile and rectal temperature were collected at the following times: 0 h (before disbudding), 2 h after disbudding, 4 h after disbudding, 6 h after disbudding, and 24 h after disbudding.

### 2.8. Statistical Analysis

All statistical analyses were performed using SAS software, version 9.4 (SAS Institute Inc., Cary, NC, USA). The experiment was analyzed as a completely randomized design, with calves serving as the experimental units. Prior to the analysis of descriptive statistics, the normality of residuals was verified using the Shapiro–Wilk test in SAS PROC UNIVARIATE. Data points with studentized residuals greater than +3 or less than −3 were considered “outliers” and excluded from the analyses. The homogeneity of variances was compared using the Levene test.

Data that met the assumptions of residual normality and homogeneity of variances were analyzed using PROC MIXED. For data aggregated to create a single measure, the following model was applied:

Yij = μ + Ti + Eij

where Yij represents the response variable, μ is the overall mean, Ti is the treatment effect (UD), and Eij is the random (residual) effect.

Growth performance data, blood metabolites, cell count, fecal score, and inflammatory profile were analyzed as repeated measures over time, with the sampling day as a fixed effect. The model incorporated treatment as a fixed effect and was represented as:

Yijk = μ + Ti + Sk + TSik + Eijk

where Yijk denotes the response variable, μ is the overall mean, Ti is the treatment effect (UD), Sk is the fixed effect on the sampling day, TSik is the interaction between treatment and sampling day, and Eijk is the residual effect. Among the 15 covariance structures tested, the one that best fit the statistical model was selected based on the lowest corrected Akaike’s Information Criterion (AIC) value.

The odds ratio (binomial logit) of clinical problems, digestive problems, and pneumonia during the experimental period was analyzed by logistic regression using the GLIMMIX procedure, with the control group as the base for comparison. For all analyses, differences were considered significant at *p* ≤ 0.05 and trends were noted at *p* > 0.05 to 0.10.

## 3. Results

Ultra-diluted treatment did not decrease fecal score, diarrhea incidence, or respiratory diseases during 75 days of treatment (*p* > 0.05; Table 1). The calves in the CON group showed a 62.5% incidence of diarrhea, while the UD group had a 41.18% incidence. It was expected that the group that received the ultra-diluted compound would have a decreased fecal score, which was not confirmed by the present data; the control group had a fecal score of 1.94, and the group that received the ultra-diluted compound had a fecal score of 2.00. The score was evaluated daily, and for data analysis, the average of the 7 days was calculated to represent the week.

There was no effect of age × treatment interaction (*p* > 0.05) or treatment effect (*p* > 0.05) on feed intake; body weight; or average daily gain of heart girth, hip width, or withers height (Table 2). However, animal age (time) affected feed intake, heart girth, hip width, and withers height (*p* < 0.0001), which was expected, since the animals were in the development phase (Table 2).

The ultra-diluted treatment and the age x treatment interaction did not affect the erythrogram variables; however, a trend was observed for platelets (*p* = 0.06; Table 3), in which calves fed with ultra-diluted treatment showed an increase in platelet count compared to the control group. Animal age had a significant effect on all leukogram variables (*p* < 0.0001).

Blood urea nitrogen, aspartate aminotransferase, gamma-glutamyl transferase, total protein, albumin, globulin, and creatinine concentrations did not differ between treatments (*p* > 0.05; Table 4), but they were affected by animal age (*p* < 0.0001, Table 4).

## 4. Discussion

The use of the ultra-diluted complex had no significant impact on the health aspects of the calves in this study, as indicated by the absence of effects on the incidence of diarrhea and respiratory diseases, as well as the fecal score. A diarrhea diagnosis was considered when the calf had fecal score greater than or equal to 3. In the study by [15] a study was conducted on weaned Holstein calves (83 ± 7.9 d of life; 112.5 ± 11.7 kg of body weight), evaluating the same ultra-diluted complex used in this present study. Their results revealed a reduction of approximately 20% in digestive problems in the group that received the ultra-diluted complex, contrasting with the results obtained in our current study.

A possible explanation for this discrepancy between the studies is that, at this stage, the calves were still developing their immunocompetence, which provides the capacity and intensity to mount an immune response to defend against pathogens. This may have influenced their response mechanisms to the ultra-diluted complex, given that the maturity of the immune system is typically reached at around 6 months of age [7,16].

A study by [30] evaluated the effect of an ultra-diluted complex as a prophylactic for diarrhea in dairy calves. The product used in their study had two compounds in common with those of the product used in the present experiment (*Colibacillinum and Podophyllum).*

Reduced incidence of diarrhea by 50% compared to the control group was found, which was not confirmed by our study.

Their findings showed that the ultra-diluted complex reduced the incidence of diarrhea by 50% compared to the control group, which was not confirmed by our study. A reason for this discrepancy could be the absence of a specific compound in the ultra-diluted formulation used in the present research that may have contributed to the outcomes observed in [30]. It is important to note that, in the formulation of homeopathic products, it is common to employ a blend of compounds. This practice complicates the ability to establish direct cause-and-effect relationships.

The starter concentrate intake, BW, daily weight gain, heart girth, hip width, and withers height were not affected by ultra-diluted complex supplementation. However, all variables increased over the weeks according to already-expected body development.

Despite the high incidence of diseases between groups, with an average of 51.8% for diarrhea and 45.7% for respiratory diseases, higher values than those recommended for the milk-feeding period, the animal performance can be considered satisfactory, with an average daily gain (ADG) of 764 g/d [31].

Although there was no difference between treatments, these findings are relevant. Due to the high incidence of diseases, low performance was expected due to the negative impacts of diarrhea and respiratory diseases on animal welfare, growth performance, and productive performance, as well as an increase in mortality rates [28,32]. The satisfactory ADG performance despite the high incidence of diseases can be associated with the daily monitoring of animals through health score and temperature, which allowed the early diagnosis of diseases and rapid treatment, generating good prognoses when compared to late diagnosis. Both studies [28,32] also reported the importance and benefits of early diagnosis.

Blood parameters are important indicators of nutritional or metabolic problems and assist in the diagnosis of many diseases [33,34]. The ultra-diluted complex did not affect the hematology parameters during the milk-feeding period, except for platelets (tendency) in the control group, in which an 11% greater count was observed in the ultra-diluted complex group compared to the control.

Such findings are not in agreement with the results obtained for diarrhea incidence, and although animals that received the ultra-diluted complex presented 21.32% fewer diarrhea cases compared to the control group, they were not statistically different, probably due to the low number of experimental units enrolled. This change in platelet count can be considered an isolated case without cytopenias. Thus, the most cautious strategy for this study is to discard this possible effect to reach a conclusion on the effectiveness of this ultra-diluted complex, but for further studies, this effect must be further exploited. In this work, blood tests were performed every 21 days (due to the routine of the commercial farm), the most appropriate being weekly or after 3 days of diarrhea symptoms, as described [35].

Ultra-diluted complex supplementation did not affect liver metabolites or enzymes, demonstrating that it does not interfere with detrimental liver and kidney parameters, which it is important to monitor when testing new drugs and additives.

The concentrations of circulating immunoglobulins, especially IgG and IgA, are important indicators of immune function [36]. Immunoglobulin G is the main antibody mediating humoral immunity and is the most abundant antibody in serum [37], while IgA is the main antibody in exocrine secretions [38].Therefore, an expected outcome was an increase in these antibodies in animals that received the homeopathic compound, which could indicate a more active immune system, both at time zero before the natural challenge of disbudding and over time. However, this increase was not observed in the present study.

Acute phase proteins have been used as nonspecific markers of the inflammatory profile, with haptoglobin being one of the main markers for ruminants; it is frequently used in studies evaluating the disbudding process [39,40]. However, no effect was observed with the use of the ultra-diluted compound, nor over time.

Transferrin, albumin, and ceruloplasmin, also known as acute phase proteins, were not affected by the homeopathic compound. Regarding time, transferrin showed altered behavior, with an increase 4 h after dehorning, coinciding with an increase in the rectal temperature of the animals, which returned to basal levels 24 h after the procedure. Albumin also showed changes two hours after disbudding, returning to normal 4 h later.

These analyses were performed taking advantage of the stressor of disbudding, which is a stressful and very common management practice in dairy farming [41], to try to understand a possible mechanism of action of homeopathic compounds in addition to the more classic parameters evaluated for calves, as some metabolites are altered only when the organism faces an immunological challenge. To date, this is the first study to explore the effect of this ultra-diluted compound on disbudding.

Our study focused on presenting results related to health, performance, biochemical and hematological parameters, and disease incidence, which are considered essential in studies involving calves and the evaluation of new compounds. Although we did not find significant differences, we believe that our results provide a valuable starting point and reference for future studies.

A strong point of this project is the rigorous control of factors that could interfere with response variables. This includes the exclusion of animals from dystocic births, which have higher morbidity and mortality rates [3], and the inclusion of only animals with efficient passive immunity transfer, a crucial factor for calf performance and immune response [1,17]. This rigorous control allowed for an analysis with a simpler statistical model and homogeneous experimental units, providing greater reliability to the results presented.

Despite the rigorous local control and homogeneity of the experimental units, it is important to highlight some limitations of this study. In the analysis of disease incidence, a larger number of animals would be more appropriate, suggesting that the results should be interpreted with caution. Moreover, this study evaluated the effects of multiple compounds simultaneously, complicating the analysis of individual effects. This pattern is common in recent homeopathy studies [15,30,42] where multiple compounds are combined. However, to advance knowledge and increase the reliability of results in the field of homeopathy, more specific designs with separate evaluation of compounds should be considered. This approach can improve the reproducibility of results and their acceptability by the scientific community.

## 5. Conclusions

The ultra-diluted complex at this dosage did not effectively reduce the incidence of diseases or improve performance during the pre-weaning phase. However, our findings provide a valuable foundation for future research investigating the potential effects of ultra-diluted complexes on the metabolism and performance of dairy calves.

## Figures and Tables

**Table 1:** Fecal score disease incidence in pre-weaned calves supplemented or not with ultra-diluted complex.

Variable	Treatment ^1^	
	CON	UD
Diarrhea		
Fecal score	1.94	2.00
Rate, %	62.5	41.18
Odds ratio, (95 IC)	Baseline	0.42 (0.098–1.801)
*p*-Value		0.2334
Pneumonia		
Rate, %	56.25	35.29
Odds ratio, (95 IC)	Baseline	0.42 (0.099–1.82)
*p*-Value		0.2387

^1^ Control = calves that received placebo treatment 5 mL/d; ^1^ ultra-diluted complex = 5 mL/d.

**Table 2:** Feed intake and performance of pre-weaned calves supplemented or not with ultra-diluted complex.

Variable	Treatment ^1^		SEM	*p*-Value		
	CON	UD		Treat	Time	Treat × Time
Feed intake						
Milk replacer, L/d	5.73	5.73	0.08	0.93	<0.001	0.98
Starter, g/d	397.49	338.29	0.08	0.08	<0.001	0.81
Mean BW, kg						
Initial	36.81	36.06	1.27	0.68	.	.
Final	90.69	95.76	4.46	0.43	.	.
Average of the total period	56.69	59.88	3.25	0.49		.
Weight gain, kg/d	0.76	0.77	0.04	0.81	.	.
Body measures, cm						
Withers height	73.22	73.66	0.29	0.30	<0.001	0.97
Hip width	22.34	22.50	0.14	0.42	<0.001	0.99

^1^ Control = calves that received placebo treatment 5 mL/d; ^1^ ultra-diluted complex = 5 mL/d.

**Table 3:** Hematologic parameters of pre-weaned calves supplemented or not with ultra-diluted complex.

Variable	Treatment ^1^		SEM	*p*-Value		
	CON	UD		Treat	Time	Treat × Time
RBC (×$10^{12}$/L)	6.69	7.00	0.20	0.26	0.02	0.57
HGB (g/L)	104.94	108.82	2.98	0.36	0.54	0.50
HCT (L/L)	0.33	0.34	0.01	0.34	0.53	0.26
MCV (fL)	50.83	49.58	0.81	0.28	<0.001	0.21
MCH (×$10^{12}$ g)	15.94	15.72	0.25	0.53	<0.001	0.54
MCHC (g/L)	318.41	317.33	3.09	0.81	<0.001	0.17
WBC (×$10^{9}$/L)	8.57	7.36	0.51	0.11	<0.001	0.92
Neutrophils (×$10^{9}$/L)	3.69	3.48	0.01	0.12	<0.001	0.67
Lymphocytes (×$10^{9}$/L)	4.39	3.45	0.01	0.13	<0.001	0.66
Monocytes (×$10^{9}$/L)	0.44	0.38	0.00	0.95	0.12	0.94
PLT (×$10^{9}$/L)	251.50	279.30	10.37	0.06	0.03	0.98

RBC: Red blood cell count; HCT: hematocrit; HGB: hemoglobin concentration; MCV: mean corpuscular volume; MCH: mean corpuscular hemoglobin; MCHC: mean corpuscular hemoglobin concentration; WBC: white blood cell count; neutrophils; lymphocytes; monocytes; PLT: platelets. ^1^ Control = calves that received placebo treatment 5 mL/d; ^1^ ultra-diluted complex = 5 mL/d.

**Table 4:** Blood metabolites of pre-weaned calves supplemented or not with ultra-diluted complex.

Variable	Treatment ^1^		SEM	*p*-Value		
	CON	UD		Treat	Time	Treat × Time
Urea, mg/dL	21.12	21.56	1.58	0.85	<0.001	0.84
AST ^2^, U/L	45.75	42.70	2.43	0.38	<0.001	0.50
GGT ^3^, U/L	109.14	99.68	13.05	0.61	<0.001	0.64
Total protein, g/dL	5.84	5.76	0.13	0.65	<0.001	0.43
Albumin, g/dL	2.80	2.78	0.03	0.65	<0.001	0.07
Globulin, g/dL	3.04	2.98	0.13	0.74	<0.001	0.42
Creatinine, mg/dL	1.12	1.12	0.04	0.9500	<0.001	0.5664

^1^ Control = calves that received placebo treatment 5 mL/d; ^1^ ultra-diluted complex = 5 mL/d; ^2^ aspartate-aminotransferase; ^3^ gamma-glutamyltransferase. The inflammatory profile, Immunoglobulin A, Immunoglobulin G, ceruloplasmin, transferrin, albumin, haptoglobin, and rectal temperature after disbudding challenge were not affected by the treatments (*p* < 0.0001, Table 5).

**Table 5:** Inflammatory profile of pre-weaned calves supplemented or not with ultra-diluted complex.

Variable ^1^	Treatment ^2^		SEM	*p*-Value		
	CON	UD		Treat	Time	Treat × Time
IgA (mg/dL)	149.02	133.36	6.61	0.10	0.48	0.71
IgG (mg/dL)	1021.67	1108.24	0.40	0.13	0.11	0.73
Albumin (g/L)	4623.24	4559.41	48.97	0.35	0.02	0.77
Ceruloplasmin (mg/dL)	57.91	54.62	2.30	0.31	0.30	0.89
Haptoglobin (mg/dL)	26.05	24.35	1.34	0.21	0.09	0.91
Transferin (mg/dL)	372.29	370.47	11.29	0.91	<0.001	0.77
Rectal temperature (°C)	39.13	39.14	0.05	0.96	<0.001	0.72

^1^ Blood samples were collected on days 0, 2, 4, and 24 h after disbudding. ^2^ Control = calves that received placebo treatment 5 mL/d; ^1^ ultra-diluted complex = 5 mL/d.

## Data Availability

The raw data from this study will be made available by the authors (corresponding author) upon request to any qualified researcher.
